# TXNL1 has dual functions as a redox active thioredoxin-like protein as well as an ATP- and redox-independent chaperone

**DOI:** 10.1016/j.redox.2023.102897

**Published:** 2023-09-26

**Authors:** Attila Andor, Mahendravarman Mohanraj, Zsuzsanna Anna Pató, Katalin Úri, Beáta Biri-Kovács, Qing Cheng, Elias S.J. Arnér

**Affiliations:** a Department of Selenoprotein Research and the National Tumor Biology Laboratory, National Institute of Oncology, 1122, Budapest, Hungary; b Division of Biochemistry, Department of Medical Biochemistry, Karolinska Institutet, SE-171 77, Stockholm, Sweden

**Keywords:** Thioredoxin, Chaperone, Insulin, Disulfide, Reduction

## Abstract

TXNL1 (also named TRP32, for thioredoxin related protein of 32 kDa) is a cytosolic thioredoxin-fold protein expressed in all cell types and conserved from yeast to mammals, but with yet poorly known function. Here, we expressed and purified human TXNL1 together with several Cys-to-Ser variants, characterizing their enzymatic properties. TXNL1 could reduce disulfides in insulin, cystine and glutathione disulfide (GSSG) in reactions coupled to thioredoxin reductase (TXNRD1, TrxR1) using NADPH, similarly to thioredoxin (TXN, Trx1), but with lower catalytic efficacy due to at least one order of magnitude higher *K*_*m*_ of TrxR1 for TXNL1 compared to Trx1. However, in sharp contrast to Trx1, we found that TXNL1 also had efficient chaperone activity that did not require ATP. TXNL1 made non-covalent complexes with reduced insulin, thereby keeping it in solution, and TXNL1 provided chaperone function towards whole cell lysate proteins by preventing their aggregation during heating. The chaperone activities of TXNL1 did not require its redox activity or any dithiol-disulfide exchange reactions, as revealed using Cys-to-Ser substituted variants, as well as a maintained chaperone activity of TXNL1 also in the absence of TrxR1 and NADPH. These results reveal that TXNL1 has dual functions, supporting TrxR1-driven redox activities in disulfide reduction reactions, as well as being an ATP-independent chaperone that does not require involvement of its redox activity.

## Highlights

• The thioredoxin-fold protein TXNL1 was recombinantly expressed and characterized.
• TXNL1 reduces disulfide substrates similarly to Trx1.
• TXNL1 shows a higher *K*_*m*_ for TXNRD1 than Trx1 resulting in lower catalytic efficacy.
• In contrast to Trx1, TXNL1 is also an efficient chaperone.
• The chaperone activity of TXNL1 does not require ATP nor any of its cysteine residues.
• TXNL1 has dual functions: disulfide reduction activity and chaperone properties.

## Graphical abstract

**Figure:**
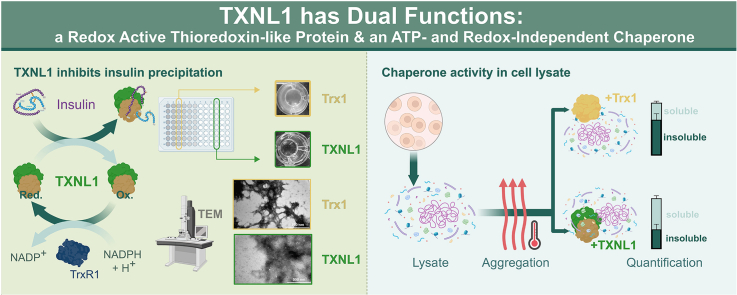

## 1 Introduction

Reduction and oxidation (redox) processes are essential for all organisms, with many important roles in maintaining cellular functions, promoting cell viability and regulating a myriad of cellular signaling pathways through redox control [[1], [2], [3]]. The thioredoxin (Trx) and glutathione (GSH) systems are the two major systems in cells controlling redox pathways through promotion of reductive processes. The Trx system includes Trx and several additional Trx-fold proteins [[4], [5], [6]], isoforms of thioredoxin reductase (TrxR, also abbreviated TXNRD), and NADPH, promoting and regulating a large number of enzymes and proteins, including peroxiredoxins, methionine sulfoxide reductases, ribonucleotide reductase and redox controlled transcription factors [[7], [8], [9]]. The GSH system, promoting reducing power in parallel with the Trx system, utilizes the GSH/GSSG redox couple that is kept reduced by the NADPH-dependent activity of GSH reductase (GR), donating electrons to isoforms of glutaredoxin (Grx) and other GSH-dependent enzyme that propel the many functions of the GSH system [[10], [11], [12]].

The Trx-fold is one of the most used and versatile protein structures in nature [[4], [5], [6]], with many members of this protein family still being only superficially studied or understood. One such protein is Thioredoxin-like protein 1 (TXNL1; also named TRP32, for thioredoxin related protein of 32 kDa) that is a thioredoxin reductase 1 (TrxR1)-dependent protein ubiquitously expressed and found in the cytosol of most or all tissues, from single-cellular organisms to mammals.^1^ TXNL was first discovered in 1998 as a novel cDNA clone [13] and the recombinant protein was found to have Trx-like insulin disulfide reduction activity [14]. Later several groups found that TXNL1 associates with the proteasome through binding to Rpn11 of either the 19S [15] or 20S [16] particles, as well as the mature 26S proteasome of both human [17,18] and yeast [19]. The functional roles of TXNL1, however, have remained essentially unknown.

**Fig. 1:**
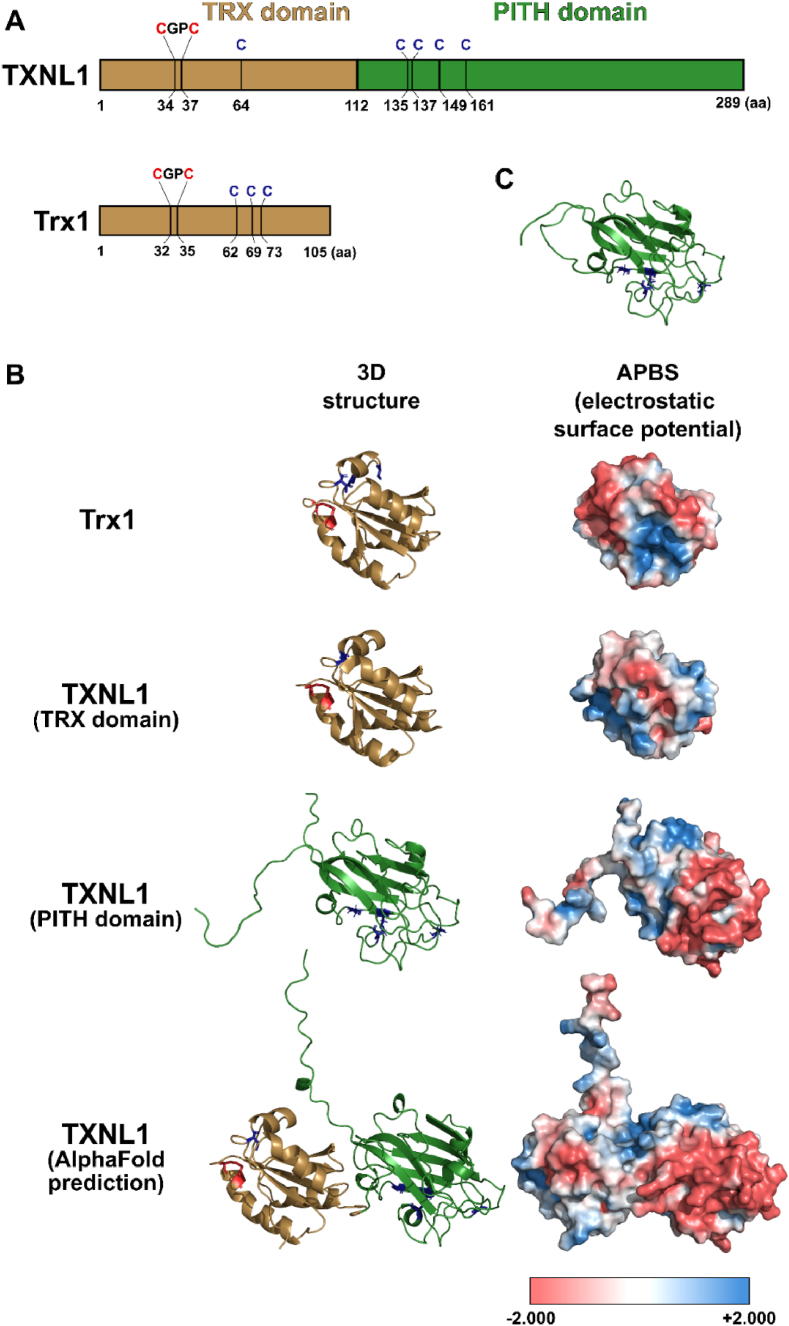
Domain organization and predicted surface features of human TXNL1 and Trx1. (A) Schematic domain organization of TXNL1 and Trx1 with their conserved active site sequence motifs (CGPC), typical of Trx proteins. Active site cysteines are highlighted and numbered in the schemes of both proteins (red), as are the additional Cys residues in both proteins (blue). (B) X-ray structures of Trx1 (PDB-structure: 1ERU) and the isolated Trx-domain of TXNL1 (PDB-structure: 1GH2), as well as the NMR-structure of the isolated PITH domain of TXNL1 (PDB-structure: 1WWY) together with the full-length structure of TXNL1 as predicted by AlphaFold (AF-O43396-F1), shown in the same orientation and scale using PyMol software and with the Cys residues shown as balls and sticks and color marked as in (A). In the right column of panel (B), the predicted electrostatic surface potentials of the proteins or domains are shown in the same orientation as in the left column, as visualized using the APBS plugin of PyMol; blue color is positive surface potential and red is negative. (C) Video (available in the online version) demonstrating the high flexibility of the C-terminal tail of the PITH domain of TXNL1 as found in prior NMR analyses (PDB structure: 1WWY). (For interpretation of the references to color in this figure legend, the reader is referred to the Web version of this article.)

TXNL1 has two distinct domains, with an N-terminal Trx-fold having a typical Trx-like active site motif (-Cys-Gly-Pro-Cys-) fused with an additional C-terminal PITH domain (formerly named DUF1000) (Fig. 1A). The PITH domain abbreviation stands for “Proteasome-Interacting Thioredoxin” and seems to be a specific feature of TXNL1 proteins from diverse organisms.^2^ The PITH, having predominantly a β-sandwich structure, promotes binding of TXNL1 with the proteasome [18] and the solution NMR structure of the isolated PITH domain also reveals a plausible protein binding motif followed by a highly flexible C-terminal tail [20]. The crystal structure of the isolated Trx-fold domain has been solved and shown to be similar in structure to Trx1, but with another surface charge distribution and more positively charged that Trx1 around the active site [21]. The full length TXNL1 protein has been difficult to crystallize, perhaps due to the high mobility and hydrophobicity of the C-terminal domain. However, using AlphaFold prediction with full-length TXNL1 the C-terminal tail is predicted to protrude out towards solvent and back over the Trx-domain (Fig. 1B). Together with surface charge analyses using PyMol (Fig. 1B), the hydrophobic features of the PITH domain become easily visualized (Fig. 1B). When also considering that the C-terminal tail of TXNL1 is highly flexible, as found in the NMR analyses of this domain and easily visualized (Fig. 1C; video in the online version of the article), it becomes predicted that TXNL1 has a hydrophobic tail that switches between solvent exposure, likely binding to other proteins, and looping back over the Trx-domain of the protein. These features of TXNL1 should be key for guiding its biochemical properties and for distinguishing the characteristics of this protein from those of Trx1.

Although some reports have suggested that TXNL1 could be of pathophysiological importance as a link between oxidative stress and proteasome function, with TXNL1 protecting cells against glucose deprivation but not H_2_O_2_ treatment [22], and being induced by prostaglandin E_2_ [23], not much is yet known about its exact functions. When active site Cys-to-Ser mutants of TXNL1 were used in unbiased trapping experiments, the protein was found to bind to significantly more proteins in HEK293 cells compared to Trx1 (1655 vs. 379, respectively) but, interestingly, not in any evident redox dependent manner since the active site trapping mutants bound virtually the same protein partners as the wildtype enzyme or the redox inactive active site Cys-less mutant, and no obvious redox-related substrates of TXNL1 could be identified [24]. That result could perhaps be compatible with a notion that the PITH domain of TXNL1, with its hydrophobicity and postulated general protein binding features, has a capacity of broadly binding other proteins. Noteworthy, recent results of ours demonstrated that TXNL1 was the most rapidly and efficiently downregulated protein (among more than 8000 proteins analyzed in a comprehensive proteomic analyses) when cultured cancer cells were treated with auranofin [25], and others have also confirmed that TXNL1 is uniquely downregulated by auranofin treatment [26]. With auranofin being an FDA-approved anti-inflammatory gold compound inhibiting TrxR1 [25,27,28] and currently being evaluated in clinical trials for repurposing in cancer treatment (see https://www.clinicaltrials.gov), and with TXNL1 yet having poorly understood functions, we here wished to further characterize the biochemical properties of TXNL1. Our aim was to compare its enzymatic properties side by side with Trx1 in TrxR1-driven redox activity assays. Interestingly, immediately when analyzing reduction of the disulfides in insulin, which is a model assay for Trx1 [29,30], we noted that insulin reduced by TXNL1 remained in solution. This surprising finding contrasted the characteristic precipitation of reduced insulin seen upon reduction with Trx1, which is often utilized as a key feature of this assay for following Trx1 activity [29,30]. Following up upon this finding led us to the discovery that TXNL1 not only possesses Trx-like redox activity, but also has an efficient ATP- and redox-independent chaperone activity.

## 2 Materials and methods

### 2.1 Chemicals and reagents

DMSO, BSA, human insulin solution, sodium selenite, kanamycin and streptomycin were from Sigma-Aldrich. l-Glutathione oxidized (GSSG, glutathione disulfide), l-Glutathione reduced (GSH) and NADPH were from AppliChem. DTT was from VWR. EDTA was from Thomasker. l-Cystine disodium salt and IPTG were from Thermo Fisher Scientific. l-glutamine and antibiotic PEN-STREP (penicillin and streptomycin) were from LONZA. Recombinant human HSP27 protein (ab4870) was purchased from Abcam. Protein concentrations were determined using the Pierce BCA assay kit. Protease inhibitor cocktail and phosphatase inhibitor mini tablets were from Thermo Fisher Scientific.

### 2.2 Plasmids and *E. coli* strains

Recombinant human TXNL1 (Uniprot: O43396; NCBI reference sequence: NP_004777.1) was here cloned and produced for this study. The open reading frame for TXNL1 was synthetized after codon optimization for *E. coli* expression by placing an order to Integrated DNA Technologies, Inc. (IDT) with the synthetic gene subsequently cloned into the pD441 plasmid developed by us [31] to generate a fusion protein of TXNL1 linked with a His-tagged GFP-Sumo protein, named 6xHis-GFP-SUMO-Hs TXNL1 (for plasmids used here and resulting protein sequences, see Table 1). The final expression plasmid was transformed into BL21 (DE3) for protein production and transformants were grown in medium with kanamycin. Human Trx1 (Uniprot: P10599-1; GenBank: AAA74596.1; NCBI Reference Sequence: NP_003320.2) was prepared in the same manner as TXNL1, following our previously described protocol [31]. Cys-to-Ser mutants of TXNL1 were made by either site directed mutagenesis using the TXNL1 expression plasmid as template, or as synthetic genes again ordered from IDT with the respective TGC/TGT codons for Cys changed to AGC for Ser (Table 1). Recombinant Sec-containing human TrxR1 (TXNRD1_v1, TrxR1a, Uniprot: Q16881-5; NCBI Reference Sequence: NP_877419.1) was prepared as described previously [31]. The Sec content of the TrxR1 preparation used in this study was 23.6%, as determined using selenium content determination with ICP-MS kindly performed by Anna Kipp and coworkers following protocols described previously [32]. We were surprised by this low Se contents determination for the TrxR1 preparation because the methodology used to prepare it typically yields full Sec contents [31]. The explanation for the low selenium incorporation is yet unknown to us and is currently under investigation, but this was the first time the enzyme was made in our Hungarian laboratory and some unidentified confounding factor should have played a role. Still, to here report turnover numbers for the Sec-containing enzyme with Trx1 and with TXNL1, we hence correlate the measured turnover with regards to its lower Sec contents, as described in Section 2.4.

**Table 1:**
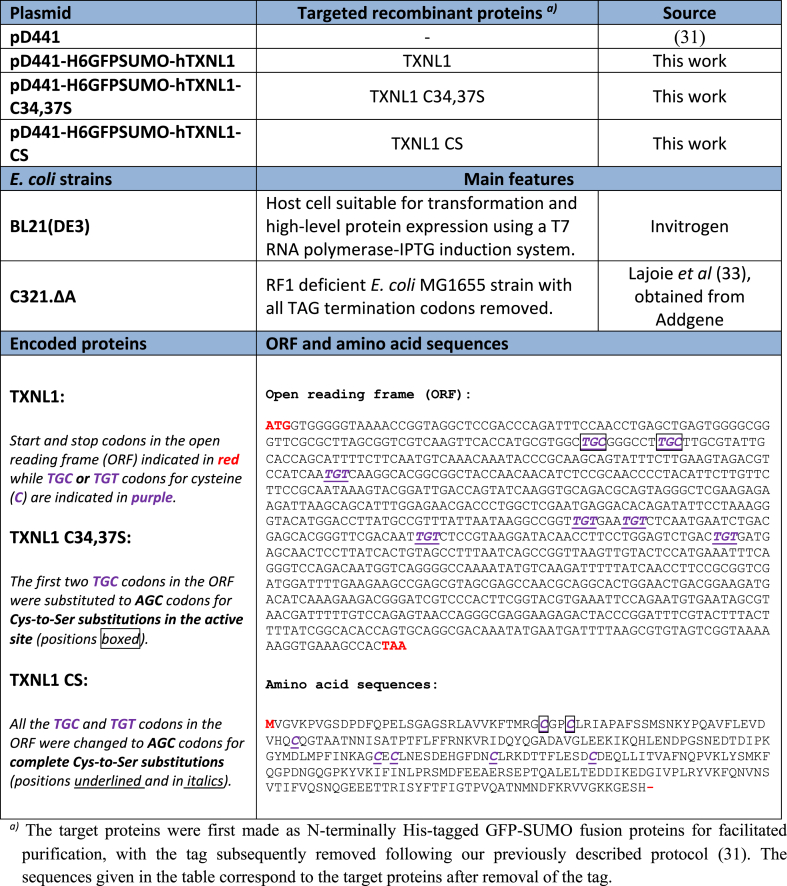
Plasmids, *E. coli* strains and human TXNL1 variants used in this study [31,33].

### 2.3 Production and purification of recombinant proteins

The recombinant Trx1, TXNL1 and its cysteine mutants were all expressed in *E. coli* BL21 (DE3), while the human selenoprotein TrxR1 was expressed in *E. coli* C321.ΔA. Expression conditions and purification of target proteins from bacterial cell lysates using affinity chromatography were basically as described previously [31]. Briefly, 40 ml aliquot of overnight cultures of the transformed bacteria was used to inoculate 2 L Terrific Broth (TB) media supplemented with the appropriate antibiotic (50 μg/ml kanamycin for pD441-constructs, or 200 μg/ml streptomycin and 50 μg/ml carbenicillin for pABC2a-HsTrxR1) in 5 L Erlenmeyer flasks. The BL21 (DE3) and C321.ΔA cells were propagated at 37 °C and 33 °C in shaking incubators, respectively. Around 4–6 h after inoculation, when the optical density of the cultures reached 1.2–1.4 at 600 nm, the temperature was decreased to 24 °C. For TrxR1 production, 5 μM sodium selenite (Na_2_SeO_3_) was added and the propagation was continued for another hour, then the protein expression was initiated by adding 0.5 mM IPTG and the culturing was continued overnight. After propagation, bacterial cells were harvested by centrifugation and suspended in binding buffer for nickel-column affinity chromatography (50 mM Tris-HCl, 250 mM NaCl, 25 mM imidazole, pH 7.5). Extraction of proteins was achieved by sonification, and the cell lysate was centrifuged. The supernatant containing the soluble fraction of proteins was loaded to HisPrep FF 16/10 column installed on an ÄKTA Explorer system (Cytiva Life Sciences). After washing out the contaminating proteins from the column, the 6xHis-GFP-SUMO-tagged target protein was eluted from the column under isocratic condition using 40% elution buffer (50 mM Tris-HCl, 250 mM NaCl, 500 mM imidazole, pH 7.5). The fusion tag was subsequently cleaved from the N-terminal part of the protein by 6xHis-ULP1 SUMO protease made in-house. The resulted mixture of the target protein, 6xHis-ULP1 and 6xHis-GFP-SUMO tag was re-loaded to the same HisPrep FF 16/10 column to bind the His-tagged, contaminating proteins to the column, and the purified non-tagged protein of interest could be retrieved in the flow-through. After buffer change and concentration, the recombinant proteins were stored in TE buffer (50 mM Tris-HCl, 2 mM EDTA, pH 7.5) containing 30% or 50% glycerol for non-selenoproteins or TrxR1, respectively. The purity of the isolated proteins was over 95% as judged by SDS-PAGE analysis.

### 2.4 Enzymatic assays, determination of kinetic parameters and exposure of free thiols

Kinetic assays were used to measure the activity of the purified recombinant human TXNL1 and Trx1 as a control, by adapting the previously described protocols [29,30] to 96-well microtiter plates. Assays for activities of TXNL1 or Trx1 as driven by TrxR1 and NADPH were coupled with the reduction of the disulfides in insulin, l-cystine or glutathione disulfide (GSSG), and were determined by following the consumption of NADPH through the decrease in absorbance at 340 nm. The molar absorbance of NADPH in this setup was determined using a standard curve of NADPH made in water with the same volume as in the enzyme assays, with the initial linear rates of absorbance change during the first 10–15 min used for calculations of turnover. Typically, assays were carried out with 300 μl total volume in 50 mM Tris-HCl, 2 mM EDTA, pH 7.5, including 300 μM NADPH, 10 nM TrxR1, and 10 μM of either TXNL1 or Trx1, coupled with 160 μM insulin, 250 μM l-cystine, or 1 mM GSSG as terminal electron acceptors. All activity assays were performed with 1 min time interval reads at 25 °C using a microplate reader (Techan, Infinite M Nano), with reaction mixtures without proteins (TXNL1 or Trx1) serving as background references.

For determination of kinetic constants, the Trx-fold protein concentrations were varied as indicated and enzymatic parameters were calculated using Graphpad Prism (v.8.0 GraphPad software, San Diego, CA, USA) after plotting turnover versus substrate concentration followed by Michaelis–Menten fit with nonlinear regression. All turnover numbers were corrected for the selenium content of the used preparation of TrxR1 (23.6%) meaning that the molar rate of NADPH consumption per minute was divided by the molar concentration of TrxR1 used in the assay, subsequently divided by 0.236 to yield the determined turnover numbers (min^−1^).

For determination of free thiols upon reduction of insulin by Trx1 or TXNL1, the components of the insulin assays were used at concentrations to ensure that complete insulin reduction would be possible. This was done by using combinations of components, as indicated, at the following concentrations: NADPH, 1 mM; insulin, 180 μM; TrxR1, 200 nM (containing 23.6% selenium) and Trx1 (20 μM) or TXNL1 (20 μM). The reactions were carried out in TE buffer (50 mM Tris-HCl, 2 mM EDTA, pH 7.5) at room temperature for 2 h. The amount of free thiols upon the end of reaction were subsequently determined by reaction with DTNB under denaturing conditions, using 30 μl sample added to 300 μl of 1 mM DTNB and 6 M guanidine-HCl in 50 mM Tris-HCl (pH 8.0). Absorbance was subsequently measured at 412 nm (resulting from TNB^−^ anions released upon reaction of DTNB with free thiols) and concentrations of thiols were calculated using a standard curve made with GSH at the same conditions.

### 2.5 Gel filtration

To study possible formation of TXNL1-insulin complexes in the insulin reduction assay, reaction mixtures (0.5 ml) of insulin reduction reactions (here scaled up to 2 ml total volume, with final concentrations of all reagents as above) were separated by gel filtration after a reaction time of 300 min. Control reactions were performed omitting or including components as indicated. For the chromatography, a Superdex 75 Increase 10/300 GL column (Cytiva 17-5174-01), suitable for separation of globular proteins in the molecular range of 3–70 kDa, with an ÄKTA Explorer system (Cytiva Life Sciences) and employing 100 mM NaCl containing 25 mM Tris-HCl buffer (pH 7.5) as mobile phase. Supernatants of insulin reduction mixtures were used as samples, as generated by centrifugation (20,000×*g*, 10 min, 4 °C). 500 μl was injected from each centrifugated precipitate-free reaction mixture, and the chromatography was performed with eluent flow rate of 0.5 ml/min at 4 °C with continuous measurement of absorbance at 280 nm.

### 2.6 Transmission electron microscopy (TEM)

TrxR1-coupled insulin assays were carried out as indicated in 2.4, using TrxR1 (10 nM), NADPH (300 μM), insulin (160 μM) and either Trx1 or TXNL1 (10 μM), which was allowed to proceed for 5 h, followed by TEM analysis performed as described previously [34]. The DTT-reduced insulin was prepared similarly, but using 0.5 mM DTT in place of TrxR1, NADPH and Trx-fold protein. Shortly, aliquots (150 μl) of each reaction mixture were diluted 4-fold with Tris-HCl buffer (pH 7.5) and from this, 5 μl aliquots were placed on Formvar coated nickel (Agar Sci., Essex, UK) electron microscopy grids. After drying, the grids were fixed with modified Karnovsky fixative reagent for 10 min at room temperature and then rinsed three times with Milli-Q distilled water. Finally, the grids were counterstained using 2% uranyl acetate for 5 min. Samples were analyzed under magnifications of 120,000×, using an excitation voltage of 120 kV on a JEOL JEM 1011 transmission electron microscope. Images were obtained using a CCD camera (Morada; Olympus) and iTEM software (Olympus).

### 2.7 Cell cultures and preparation of cell lysates

Human A549 (lung carcinoma) and AGS (gastric carcinoma) cell lines were cultured in RPMI-1640 medium containing 2 mM l-glutamine. HEK293T cells were cultured in Dulbecco's Modified Eagle's Medium (containing 4.5 g/L glucose, Lonza). In all cases, the cell culture media were supplemented with 10% FBS (Sigma), 100 U/mL penicillin and 100 μg/ml streptomycin, and 100 nM sodium selenite as selenium source. Cells were kept in logarithmic growth phase at 37 °C in humidified air containing 5% CO_2_. For preparation of lysates, cells were harvested at a confluence of ∼80% using TrypLE (Gibco) and resuspended in lysis buffer (50 mM Tris-HCl, 5 mM EDTA, 150 mM NaCl, 1% Triton X-100) including Protease Inhibitor Mixture Complete (Thermo Fisher Scientific), followed by three rapid freeze/thaw cycles. Lysates were cleared by centrifugation and stored at −80 °C until analyzed.

### 2.8 Immunoblot analyses

For immunoblot analyses of purified proteins or cell lysates, proteins were separated on SDS-PAGE (with or without DTT as indicated in the figures) and subsequently blotted to nitrocellulose membrane (Invitrogen) with Bio-Rad Trans-Blot® Turbo™ (25 V, 7 min). Equal loading of cell lysate proteins (30 μg per lane) was confirmed using Ponceau staining (Sigma), while chromatography fractions were instead loaded with equal volumes in each lane. Blocking was done with 5% powdered milk (Carl Roth) and 0.5% BSA (Bovine Serum Albumin, Sigma-Aldrich) in TBS-T (Tris Buffered Saline (Bio-Rad) with 0.5% Tween 20, Sigma-Aldrich). Primary antibodies were obtained from either Santa Cruz (SC) or Cell Signaling Technology (CST): anti-Trx1 (SC catalog number: 166393; 1:500), anti-TXNL1 (SC: 515218; 1:1000), anti-TrxR1 (CST#6925, 1:1000), anti-HSP27 (CST#2404, 1:1000), anti-insulin B chain (SC: 377071; 1:1000) and anti-β-actin (SC: 47778, 1:1000). The membranes were incubated overnight with the primary antibodies (diluted in TBS-T), then after washing, the corresponding HRP-conjugated secondary antibodies (Dako, P-0447, 1:8000 or Dako, P-0399, 1:3000) were added to the membranes for 1 h at RT (diluted with 5% powdered milk and 0.5% BSA in TBS-T). After addition of ECL substrate (BR1705062), chemiluminescent signals were detected using a Bio-Rad ChemiDoc XRS scanner.

### 2.9 Inhibition of protein aggregation in cell lysate

The chaperone activities of recombinant human HSP27, TXNL1 or Trx1 proteins on bulk proteins were assessed by analyzing their ability to prevent temperature-induced aggregation of proteins from cell lysates using SDS-PAGE followed by a Coomassie blue staining method as described previously [35]. Briefly, cell lysates were incubated for 90 min at 45 °C in a water bath at a concentration of 1.0 mg/ml with or without addition of either TXNL1, Trx1 or HSP27 (with final concentrations between 0 and 10 μM, as indicated) in PBS containing 1 mM DTT. After incubation for 90 min at 45 °C, samples were centrifuged for 15 min at 8600×*g* to separate soluble and insoluble fractions. Insoluble pellets were washed twice with ice cold PBS, and resuspended in lysis buffer. Equal volumes of each soluble and insoluble fractions were mixed with 2x Laemmli buffer and boiled for 10 min. All fractions were subsequently subjected to SDS-PAGE and the resulting gels were stained with Coomassie blue dye. Gels were scanned with a ChemiDoc XRS Imaging System (Bio-Rad) and the amounts of protein in soluble *vs.* insoluble fractions were quantified using ImageJ software. The complete lanes were selected for these analyses, with a background subtracted using the “Substrate background” tool of the software and the band borders from the top to bottom were selected using the rectangle tool for each analyzed lane. Thereby the average grey values were obtained, and subsequently the mean grey values of bands corresponding to addition of HSP27 or Trx1 or TXNL1 added (measured accordingly) were subtracted from these values. All experiments were repeated at least three times and the mean ± S.D. were calculated. The raw densitometry values were normalized to the total amount of protein (soluble and insoluble), which was set to 100%.

## 3 Results

### 3.1 Redox activities of TXNL1 compared to Trx1

**Fig. 2:**
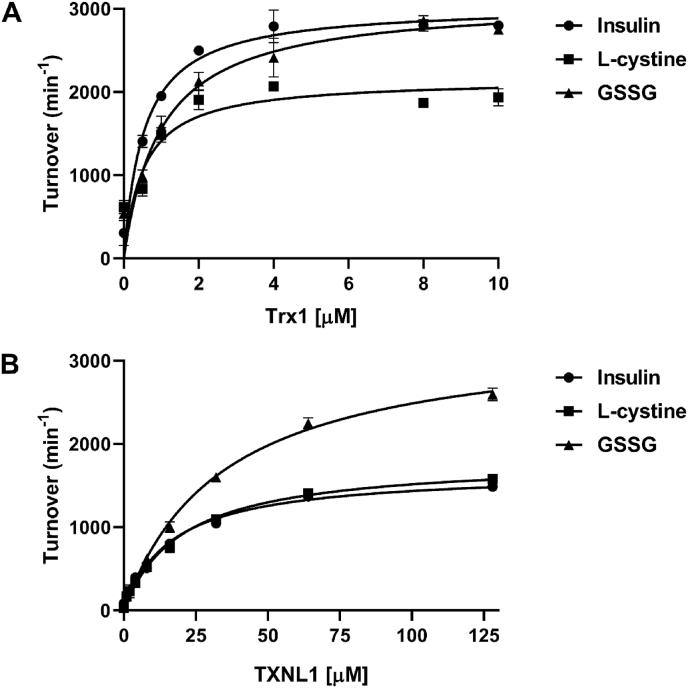
Michaelis-Menten plots with human TXNL1 and Trx1 using insulin, l-cystine and GSSG as substrates. The initial reaction rates in reduction of either insulin (160 μM), l-cystine (250 μM) or GSSG (1 mM) were determined in assays coupled with 10 nM TrxR1 and 300 μM NADPH using increasing concentrations of either ***A****)* Trx1 or ***B)*** TXNL1, as indicated. Turnover was calculated as mol NADPH consumed per mol Se-containing TrxR1. For further experimental details, see the methods description. Values are average from three independent enzyme assays each time run with single samples and error bars show ± S.D. For the resulting calculated kinetic parameters, see Table 2.

To characterize TXNL1 redox activities in comparison with Trx1, the purified recombinant proteins were first used side-by-side in typical TrxR1-driven substrate reduction assays, determining the Michaelis-Menten kinetic parameters of TrxR1 using either insulin, l-cystine or GSSG as terminal electron acceptors for either TXNL1 (Fig. 2A) or Trx1 (Fig. 2B). This showed that the K_m_ for TXNL1 was at least one order of magnitude higher than for Trx1 while the turnover numbers were comparable, thus resulting in much worse catalytic efficacy (*k*_cat_/*K*_m_) for TXNL1 compared to Trx1, although both proteins were clearly capable of reducing all three substrates (Fig. 2 and Table 2.).

**Table 2:** **Kinetic parameters of TXNL1 and Trx1.** These kinetic parameters in reduction of insulin, l-cysteine and GSSG were calculated from the data shown in Fig. 2 using the Michaelis-Menten fit with nonlinear regression of the GraphPad Prism software.

	TXNL1	Trx1
***Insulin***		
*k*_cat_ (min^−1^)	1670 ± 50	3237 ± 378
*K*_m_ (μM)	16.5 ± 1.3	1.4 ± 1.5
*k*_cat_/*K*_m_ (min^−1^ μM^−1^)	102	2294
L**-cystine**		
*k*_cat_ (min^−1^)	1820 ± 86	2175.2 ± 80
*K*_m_ (μM)	20 ± 2.5	0.8 ± 0.5
*k*_cat_/*K*_m_ (min^−1^ μM^−1^)	92	2868
**GSSG**		
*k*_cat_ (min^−1^)	3329 ± 123	3102.4 ± 51
*K*_m_ (μM)	34 ± 1.7	1.0 ± 0.7
*k*_cat_/*K*_m_ (min^−1^ μM^−1^)	98	3118

### 3.2 Chaperone activity of TXNL1 during insulin reduction in vitro

**Fig. 3:**
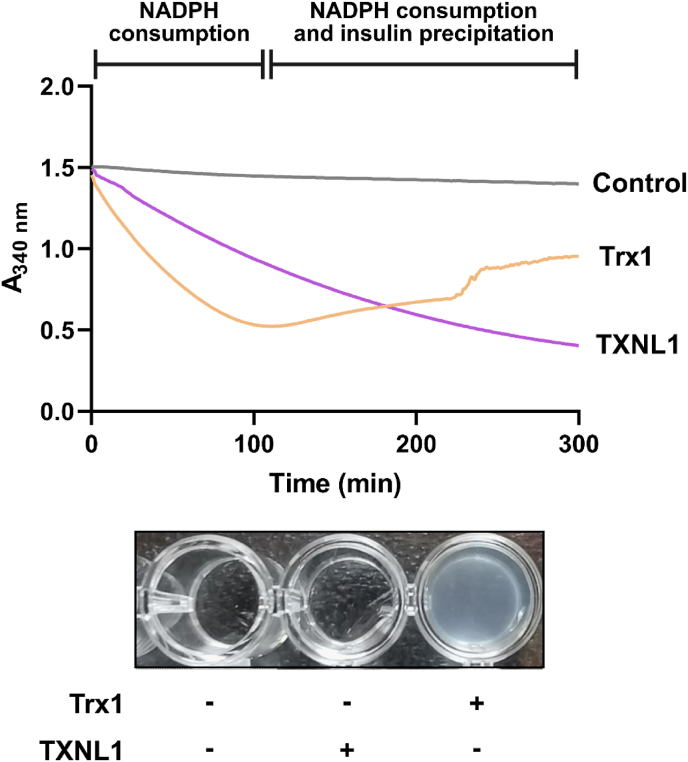
Different behavior of insulin precipitation when reduced by Trx1 or TXNL1. In the upper part of the figure raw absorbance traces at 340 nm are shown from reactions with Trx1- (orange) and TXNL1- (purple) driven insulin reduction assays ran in parallel. Both reaction mixtures contained 300 μM NADPH, 10 nM TrxR1 and either 10 μM Trx1 or TXNL1, with the control reaction (grey) containing all components listed except for a Trx-fold protein. As is clear from the traces, inclusion of both Trx1 and TXNL1 yield an initial decrease of absorbance at 340 nm that is due to NADPH consumption, while in the case of the Trx-driven reaction but not with TXNL1, the absorbance starts to increase in an irregular manner at later time points due to insulin precipitation (as indicated on top). The lower part of the figure shows a photo of the corresponding microtiter plate wells after completion of the assay, illustrating the apparent turbidity due to insulin precipitation only seen after the Trx1-driven reaction. (For interpretation of the references to color in this figure legend, the reader is referred to the Web version of this article.)

**Fig. 4:**
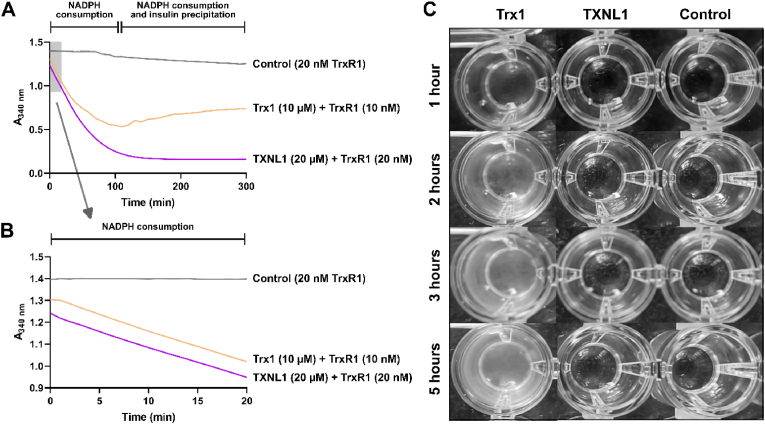
Lack of insulin precipitation upon reduction by TXNL1 is independent of reduction speed. (A) Kinetics of NADPH consumption followed as absorbance decrease at 340 nm are shown using either Trx1 and TrxR1 at 10 μM and 10 nM, respectively (orange) or TXNL1 and TrxR1 at 20 μM and 20 nM, respectively (purple), resulting in similar initial speeds of the reaction, as also shown in (B) that is a magnification of the shaded area in (A). The control reaction contained only 20 nM TrxR1 (grey) and all reactions contained 160 μM insulin and 300 μM NADPH. In (C) photos of the wells at different time points from assays ran as in (A) are shown, clearly visualizing precipitate formation only in the Trx1-driven reaction but not in that driven with TXNL1. (For interpretation of the references to color in this figure legend, the reader is referred to the Web version of this article.)

When we determined the insulin reduction with either Trx1 or TXNL1, we immediately noticed that although NADPH was clearly consumed at high initial rate in both cases (Fig. 2), there was no sign of subsequent insulin precipitation when the reaction had been catalyzed by TXNL1. This phenomenon was in major contrast to the corresponding reaction catalyzed by Trx1 and was surprising to us, because insulin typically precipitates when its disulfides are reduced. The noticeable difference between the insulin reduction reactions as catalyzed by either Trx1 or TXNL1 is best exemplified with raw kinetic traces and visualization of the resulting turbidity (or lack thereof), as shown in Fig. 3 (cf. purple and orange lines, and the opacity in the microtiter plate well of the Trx1-driven reaction). We next asked if the precipitation of the reduced insulin could have depended on the speed of the reaction. We therefore used more TXNL1 and TrxR1 in order to match the speed of the reaction with Trx1 and TrxR1 (Fig. 4A and B). This showed that although the insulin reduction proceeded with the same speed in both reactions (Fig. 4B), there was still no sign of precipitating insulin in the TXNL1-catalyzed reaction while the Trx1-catalyzed reaction in contrast at the later time points gave the typical increase in absorbance because of insulin precipitation (Fig. 4A). Photos of the reaction mixtures at different time points again clearly demonstrated the lack of precipitate in the TXNL1-driven reactions (Fig. 4C).

The phenomenon of insulin precipitating towards the later phases of reactions driven by Trx1 is typically observed, and the appearance of turbidity can even be used as a distinct feature when characterizing insulin reduction reactions catalyzed by different thioredoxins [29,30]. We were therefore surprised that this phenomenon did not occur in the reaction driven by TXNL1. Initially we reasoned that perhaps TXNL1 could only reduce one or two of the three disulfides in insulin, while Trx1 could perhaps reduce all its disulfides. That would possibly explain the lack of insulin precipitation after reduction by TXNL1, because the A and B chains of insulin likely need to be fully separated by complete disulfide reduction for its precipitation to occur. Assessing this hypothesis by determining the number of free thiols liberated from insulin by either TXNL1 or Trx1, we however found that both proteins reduced all the three disulfides in insulin (Table 3). Having thus found that TXNL1 could fully reduce insulin but nonetheless not leading to insulin precipitation, we next hypothesized that TXNL1 may have an ATP-independent chaperone activity.

**Table 3:** **Determination of free thiols (-SH) after insulin reduction using either Trx1 or TXNL1.** Insulin reduction assays were performed under conditions to ensure that all insulin (180 μM) would be possible to reduce, i.e. using higher concentrations of NADPH (1 mM), TrxR1 (200 nM) and Trx1 (20 μM) or TXNL1 (20 μM) with a long duration of assay (2 h). Reduction of 180 μM insulin would maximally result in 1080 μM thiols, which was in good agreement with the determined thiol concentrations found using either Trx1-or TXNL1-driven reduction assays (bold, first line of the table). Omitting components of the assay gave low background values, confirming validity of the assay, while omitting insulin resulted in the expected number of thiols in reduced Trx1 with five Cys residues that would yield 100 μM thiols and seven Cys residues in TXNL1 that would yield 140 μM thiols (fifth line, bold and italics). Values represent average ± S.D. from three independent experiments each run with triplicates. See text for further details.

NADPH	Insulin	Trx1-driven insulin reduction assay			TXNL1-driven insulin reduction assay		
		TrxR1 + Trx1	SH (μM)	SH/Insulin	TrxR1 + TXNL1	SH (μM)	SH/Insulin
**+**	+	+	**1087.2 ± 50.3**	**6.0**	**+**	**1056.7 ± 39.2**	**6.0**
**+**	+	–	25.5 ± 5.4	0.1			
**-**	+	+	50.8 ± 24.5	0.3	**+**	27.4 ± 26.6	0.3
**-**	+	–	23.5 ± 5	0.1			
**+**	–	+	***104.7 ± 25.5***	n.a.	**+**	***150.4 ± 18***	n.a.
**+**	–	–	31.9 ± 6.8	n.a.			

n.a. = not applicable.

### 3.3 The chaperone activity of TXNL1 does not require any of its Cys residues

**Fig. 5:**
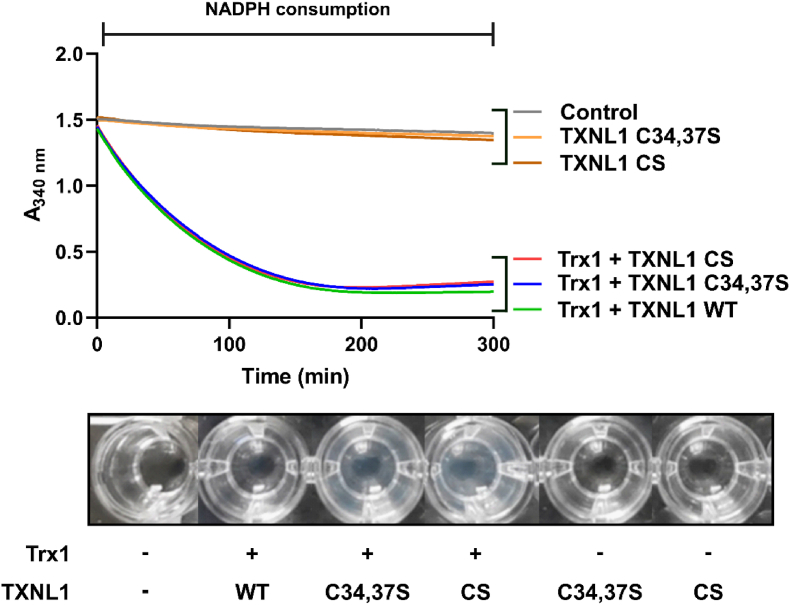
The chaperone activity of TXNL1 does not require its Cys residues. When Trx1-driven insulin reduction was performed in the presence of TXNL1 variants with or without its Cys residues mutated, the reduced insulin did not precipitate at any appreciable rate (upper part of the figure, red, blue and green absorbance traces; cf. Trx1-only trace in Fig. 3). If Trx1 was omitted from the reaction, no NADPH consumption was seen with the active site mutant (orange) or completely Cys-less variant (brown) that were identical with a control containing all components, except a Trx-fold protein (grey). All reaction mixtures contained 300 μM NADPH, 10 nM TrxR1, and TXNL1 variants or Trx1 each applied at 10 μM concentration. The lower part of the figure shows a photo of the corresponding microtiter plate wells after completion of the assay, illustrating a slight turbidity in the wells containing the Cys-mutated TXNL1 variants together with Trx1. See text for further details. (For interpretation of the references to color in this figure legend, the reader is referred to the Web version of this article.)

To assess whether a possible chaperone activity of TXNL1 would be dependent upon its redox activity or require disulfide exchange reactions, we prepared a double Cys-to-Ser substituted active site mutant, named TXNL1 C34,37S, as well as a completely Cys-less Cys-to-Ser substituted variant named TXNL1 CS (Table 1). When including any of the TXNL1 variants within the insulin assay driven by Trx1, insulin precipitation was fully prevented; this occurred although all these reactions proceeded with the same rate and were run until complete NADPH consumption, as illustrated by a low end absorbance at 340 nm (Fig. 5, upper part). This result clearly showed that TXNL1 has chaperone activity, as exemplified by the prevention of Trx1-catalyzed reduced insulin from precipitating, and that this chaperone activity of TXNL1 did not require any of its Cys residues. With the active site mutant TXNL1 C34,37S having the same effects in this assay as the completely Cys-deficient TXNL1 CS variant also showed that there is neither a requirement for the typical redox activity of TXNL1 nor for any other disulfide-dithiol exchange reactions involving the protein, in order for TXNL1 to function as a chaperone. However, a slight increase in turbidity was seen upon completion of the assays ran in the presence of the Cys mutants that was not seen in the presence of the wildtype TXNL1 (Fig. 5, lower part), suggesting that perhaps either disulfide-dithiol exchange or at least retaining an intact active site of the protein may further potentiate its chaperone properties.

### 3.4 TXNL1 makes high-molecular weight complexes with reduced insulin in a manner not requiring the cysteine residues of TXNL1

**Fig. 6:**
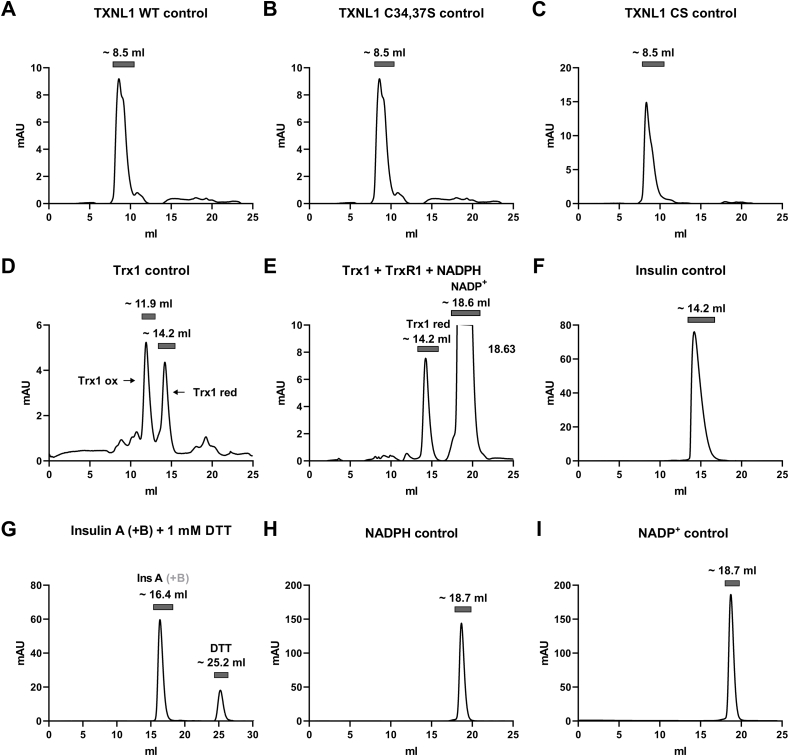
Elution profiles of individual assay components in gel filtration. The isolated components of the different insulin reduction assays were analyzed on gel filtration with measurement of absorbance at 280 nm. Here the resulting chromatograms are shown for (A) TXNL1 wildtype ("WT") protein, (B) TXNL1 C34, C37S active site mutant, (C) the TXNL1 CS completely cysteine-less mutant, (D) Trx1 with both reduced and oxidized species of the protein present, (E) a mix of Trx1, TrxR1 and NADPH resulting in purely reduced Trx1 and also seeing the NADPH/NADP^+^ peak, (F) intact insulin, (G) reduced insulin obtained after incubation with DTT (with the B chain being too small to reliably detect), H) NADPH, and I) NADP^+^. The chromatography peaks of the respective components are indicated with horizontal bars and the approximate elution volume, with the full volume of the elution given at the x-axis and absorbance at 280 nm at the y-axis given for each chromatogram. Concentrations of proteins and NADPH were as subsequently used in Fig. 7.

To assess whether TXNL1 directly binds to reduced insulin polypeptides we next attempted to isolate possible TXNL1-insulin complexes by gel filtration. For this we first determined the retention volumes of the pure assay components on a Superdex 75 Increase column using a mobile phase for mild elution conditions (100 mM NaCl, 20 mM Tris-HCl, pH 7.5). The individual components were well separated from each other in this chromatographic setup, with elution order and corresponding elution volumes as follows: TXNL1 CS, V_R_ 8.5 ml; TXNL1- C (34,37)S, V_R_ 8.5 ml; TXNL1, V_R_ 8.5 ml; Trx1, oxidized, V_R_ 11.9 ml, reduced, V_R_ 14.2 ml; Insulin, V_R_ 14.2 ml; Insulin A (+B), V_R_ 16.4 ml; NADPH, NADP^+^ V_R_ 18.7 ml (Fig. 6).

**Fig. 7:**
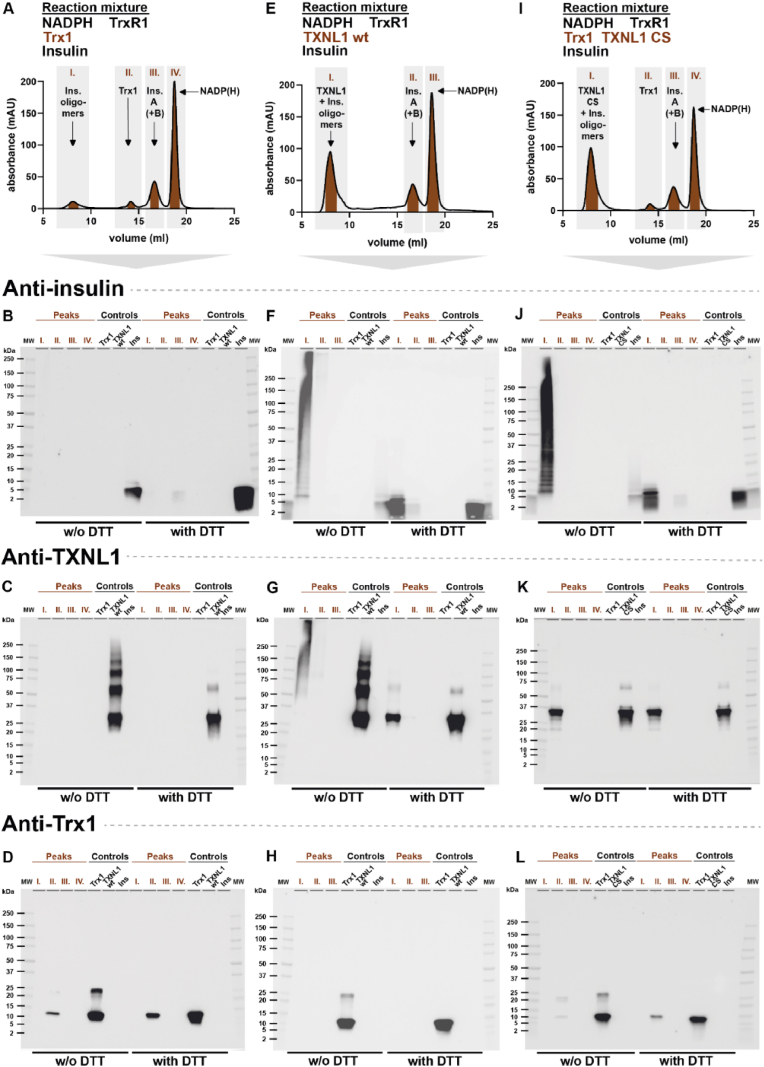
TXNL1 but not Trx1 makes high-molecular weight complexes with reduced insulin. Insulin reduction assays were performed in higher volumes and subsequently evaluated by gel filtration and with key fractions further analyzed for the presence of specific protein components using Western blots. All chromatography runs were performed as with the pure components shown in Fig. 6. Here the chromatograms after reactions with (A) solely Trx1-catalyzed insulin reduction, (E) addition of TXNL1 to the Trx1-catalyzed reaction, and (I) addition of cysteine-less TXNL1 CS to the Trx1-catalyzed reaction are shown, with identification of Peaks I, II, III and IV as indicated, elution volumes at the x-axes and absorbance at 280 nm at the y-axes. Fractions of each peak were also analyzed by Western blot analyses, blotting fractions from (A), (E) and (I) for the presence of (B), (F), (J), insulin, (C), (G), (K), TXNL1 and (D), (H), (L), Trx1, respectively, including controls with pure proteins and analyzing the fractions as well as proteins under non-reducing conditions without (“w/o”) or reducing conditions with DTT, as indicated in the figure panels. All chromatography fractions were loaded in the lanes with the same volume (2 μl each). Controls for the Western blots used were pure insulin (0.33 μg), TXNL1 (0.165 μg) and Trx1 (0.165 μg), added to their respective control lanes, as indicated.

Having established the elution behavior of the individual assay components in the gel filtration, we next performed the enzymatic reduction assays in larger volumes (2 ml) with the resulting reaction product mixtures cleared by centrifugation and subsequently analyzed on the size exclusion chromatography (Fig. 7). After the Trx1-driven reaction, negligible amounts of soluble insulin or Trx1 could be found in Peak 1, corresponding in elution volume to high-molecular weight complexes, while peaks corresponding to reduced Trx1 (Peak II), reduced insulin (Peak III) and NADP(H) (Peak IV) could be readily detected (Fig. 7A). We also analyzed the fractions corresponding to Peaks I-III with Western blots under either non-reducing (-DTT) or reducing (+DTT) conditions with comparisons to the pure control proteins under the same conditions, thereby analyzing the presence and migration behavior of insulin (Fig. 7B), TXNL1 (Fig. 7C) and Trx1 (Fig. 7D). This validated that insulin was present in Peak III, but at low amounts and it could only be seen in the Western blot when ran under reducing conditions (Fig. 7B). It is likely that most of the Trx1-reduced insulin had precipitated in the reaction mixture and was thus cleared by the centrifugation step performed before the chromatography. The Western blots also validated that Trx1 was absent in Peak I, but present in Peak II, with some dimeric Trx1 seen in the non-reducing SDS-PAGE analyses (Fig. 7D). Clearly TXNL1 was absent from all of the peaks in this chromatogram, as it had not been included in the original enzyme reaction, but the non-reducing vs. reducing conditions revealed that the control with wild type TXNL1 protein easily formed disulfide-linked multimers (Fig. 7C).

When analyzing the reaction products after the TXNL1-catalyzed insulin reduction a major absorbance was seen in Peak I (Fig. 7E), which when evaluated with Western blots revealed that both insulin (Fig. 7F) and TXNL1 (Fig. 7G) were present at high amounts in this peak, furthermore both appearing as smears in the non-reducing SDS-PAGE that could be resolved to predominantly monomeric proteins under reducing conditions (Fig. 7F and **G**, **Peak I ± DTT**). This result shows that TXNL1 and reduced insulin bind to each other into high molecular-weight complexes that elute as Peak I in the chromatography. Moreover, disulfides may have been involved in the formation of these complexes but it cannot be ruled out that the oxidation into disulfides could also have occurred during preparation of the fractions for the non-reducing SDS-PAGE. In order to evaluate whether disulfides of TXNL1 were required for the formation of its complexes with reduced insulin we thereby performed a similar chromatography experiment using the Trx1-catalyzed insulin reduction in the presence of the completely Cys-less TXNL1 CS variant (which still keeps insulin in solution under such conditions, see Fig. 5). Again, the chromatography showed a major absorbance corresponding to Peak I (Fig. 7I). The Western blot analyses verified that Peak I contained high amounts of insulin, which still easily made disulfide-linked complexes (Fig. 7J, **Peak I ± DTT**), as well as containing TXNL1 CS, which obviously could not form disulfides as it lacks cystine residues (Fig. 7K, **Peak I ± DTT**) and, importantly, Peak I from this experiment still did not contain any Trx1 and was seen only in Peak II (Fig. 7L).

**Fig. 8:**
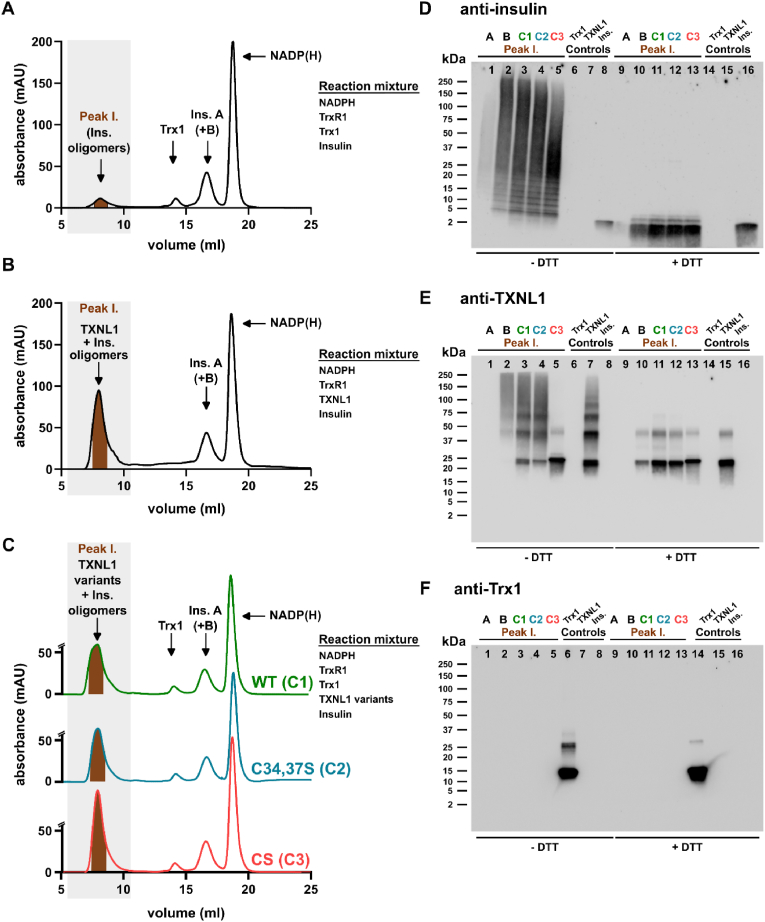
Formation of soluble high molecular weight TXNL1-reduced insulin complexes with their isolation by gel filtration do not require the cysteine residues of TXNL1. Here Peak I from the chromatograms of insulin reduction mixtures were analyzed in further detail with the proteins in the different Peak I fractions detected side-by-side in the same Western blots. In (A) the chromatogram of the sole Trx1-catalyzed reaction is shown (same as in Fig. 7A), (B) shows the chromatogram with products of the reaction carried out by Trx1 in the presence of wildtype TXNL1 (same as in Fig. 7E) and in (C) the chromatograms upon addition of the different cysteine mutants of TXNL1 are also shown as overlays for facilitated comparisons, as indicated (the final one same as in Fig. 7I). The chromatogram with addition of wildtype TXNL1 is shown in green and is abbreviated as chromatogram “C1”, that with addition of the active site mutant TXNL1 C34,37S is in blue and named “C2”, and that with addition of the full cystine-to-serine mutant TXNL1 CS is in red and named “C3”. The abbreviations relate to the labeling of the corresponding lanes in the Western blots, where aliquots (2 μl) of the Peak I fractions from the corresponding chromatograms A, B, C1, C2 and C3, were analyzed for presence and migration of (D) insulin B chain, (E) TXNL1, and (F) Trx1, along with control proteins as indicated. All conditions were the same as in Fig. 6. (For interpretation of the references to color in this figure legend, the reader is referred to the Web version of this article.)

To compare side-by side the behavior of the proteins appearing in Peak I, and to also evaluate the effects of either wild-type TXNL1 or the isolated active site mutant of the protein when added to the Trx1-assay, we also ran these enzymatic assays followed by chromatography, and compared the Western blot evaluations of all Peak I fractions collected and analyzed together in the same gels (Fig. 8). This clearly showed that TXNL1, TXNL1 C34, C37S active site mutant, as well as the completely cysteine-less TXNL1 CS mutant, all behaved the same in these chromatographic analyses (Fig. 8C). When evaluating the proteins from all the Peak I fractions of the different chromatography analyses side-by-side in the same Western blots, probing for insulin (Fig. 8D), TXNL1 (Fig. 8E) and Trx1 (Fig. 8F), several conclusions could be drawn. It became clear that when insulin has been reduced only by Trx1, the chromatography indeed showed that very low amounts of soluble high-molecular weight complexes containing insulin could be seen (Fig. 8A and D, lanes 1 and 9), likely due to its precipitation and clarification by centrifugation before the chromatography. Contrasting this finding, larger amounts of high-molecular weight complexes containing both insulin and TXNL1 could be detected using either wildtype protein added to the Trx1-driven reaction (Fig. 8B) or when using any of its cysteine mutants (Fig. 8C). In all cases the TXNL1 proteins were co-eluting with insulin, irrespective of their cysteine status (cf. Fig. 8D and E, lanes 2–5 and 103).

These results unequivocally showed that TXNL1 can make high-molecular weight complexes with reduced insulin thereby keeping the insulin in solution, and furthermore that formation of these complexes does not require any of the cysteine residues of TXNL1. It can however not be ruled out that disulfide-dithiol exchange, or retaining an intact redox active site of TXNL1, could further potentiate its binding with insulin and thereby its chaperone activity. From these experiments we could also conclude that Trx1 was not capable of forming stable high-molecular weight complexes with reduced insulin, which is a fact coinciding with the lack of ability for Trx1 to keep reduced insulin in solution.

### 3.5 TXNL1 prevents formation of fibrillar insulin as evaluated using transmission electron microscopy

**Fig. 9:**
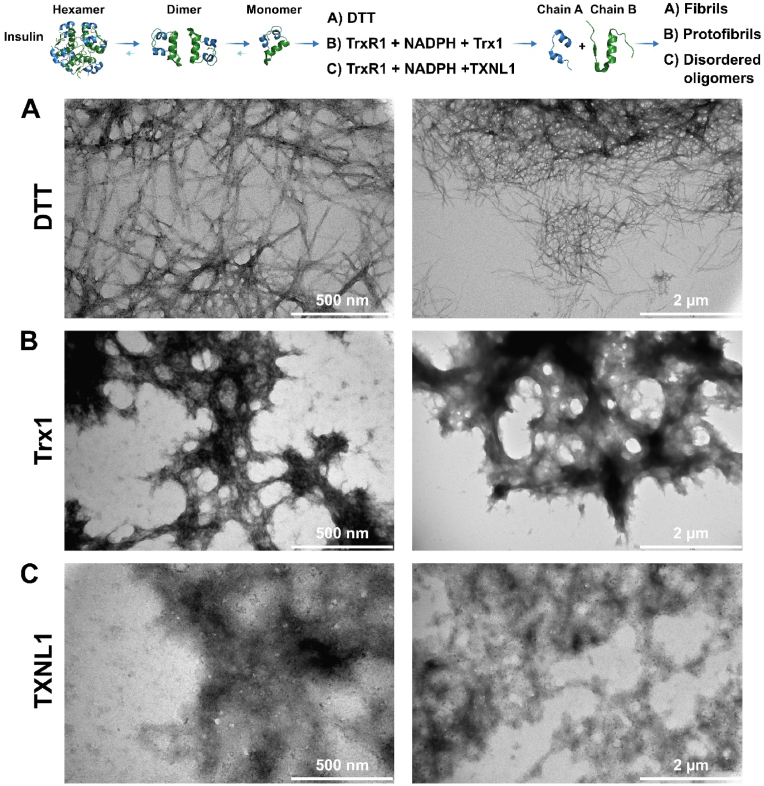
Reduction of insulin by TXNL1 prevents insulin fibril formation. On top of the figure a scheme is shown illustrating how oxidized insulin can be present as either hexamer, dimer or monomer (PDB structures visualized with the PyMol software using structures 3AIY, 6S34, 3I40 and 5E7W). When the disulfides in insulin become reduced, the A and B chains separate and then easily precipitate, thereby easily forming protofibrils, fibrils or amyloid. Here we evaluated the insulin reduction products using TEM upon either reduction using (A) 0.5 mM DTT, or enzymatically with either (B) NADPH + TrxR1 + Trx1 or (C) NADPH + TrxR1 + TXNL1, with the TEM images shown at two different degrees of magnification (see scale bar inserts). See text for further details.

Transmission electron microscopy (TEM) is a frequently used technique to visualize insulin oligomerization or fibril formation, often then reduced by DTT but also by other reductants [36,37]. TEM was here used to further distinguish the effects of insulin reduction by TXNL1 *versus* that of Trx1 or DTT with regards to insulin fibril formation (Fig. 9). In the case of insulin reduced with 0.5 mM DTT, hair-like thin braids were seen, indicating the formation of mature fibrils and amyloid insulin [36,38] (Fig. 9A). In the TEM images of the reaction mixture upon reduction of insulin with Trx1 coupled to TrxR1 and NADPH, which leads to overt precipitation of insulin similarly to that after reduction by DTT, shorter fibrils, so-called protofibrils [36,38] were detected (Fig. 9B). In the TEM analyses of the reaction mixtures using TXNL1-mediated insulin reduction, only amorphous and disordered material or oligomers could be detected (Fig. 9C). As the TXNL1-catalyzed reduction of insulin also prevents any overt precipitate formation (see above), reduction of insulin by TXNL1 thereby seems to prevent its precipitation through the capacity of TXNL1 to form soluble high molecular weight complexes with insulin (see Fig. 6, Fig. 7) that evidently also prevent formation of insulin protofibrils or amyloid. With this feature of TXNL1 being typical for a chaperone, we next assessed the possibility of TXNL1 to have general chaperone activities towards the bulk proteins in crude cell lysates.

### 3.6 TXNL1 acts as a chaperone with crude cell lysate proteins

**Fig. 10:**
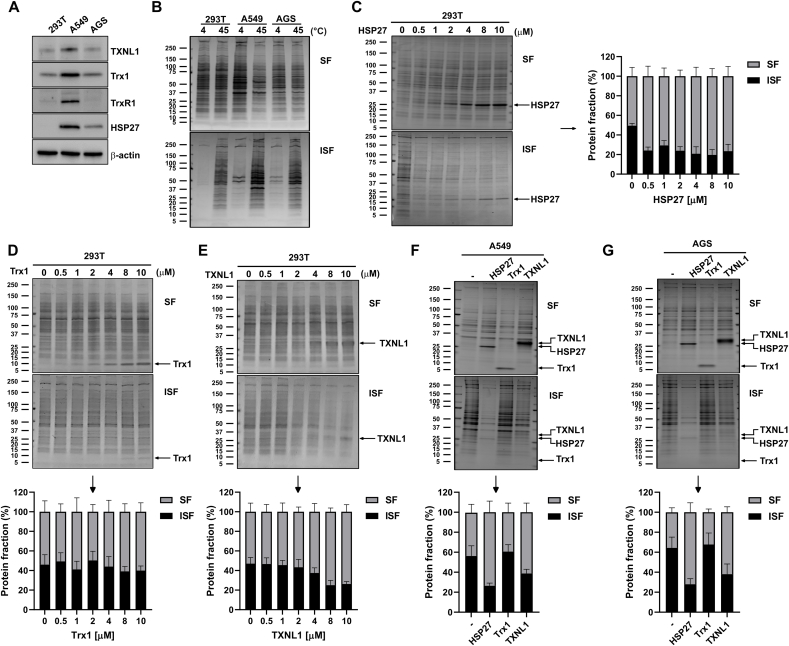
TXNL1 acts as a general chaperone as it prevents protein aggregation in heat-treated cell lysates. Cell lysates were prepared from HEK293T, A549 and AGS cells and first evaluated for (A) endogenous levels of TXNL1, Trx1, TrxR1, and HSP27 using Western blots, with β-actin used as loading control. (B) Protein of whole cell lysates (1 mg/ml) of the corresponding cell lines (indicated at the top of the figure) were incubated at 4 °C or 45 °C for 90 min in PBS containing 1 mM DTT. After incubation, soluble (SF) and insoluble fractions (ISF) were separated by centrifugation and analyzed by SDS-PAGE followed by Coomassie staining. Samples of 293T cell lysate were subsequently incubated at 45 °C for 90 min with addition of varying concentrations (0–10 μM) of (C) HSP27, (D) Trx1, or (E) TXNL1, before they were analyzed as in (B). Lysates from (F) A549 or G) AGS cells were also incubated with either HSP27 (4 μM), Trx1 (10 μM) or TXNL1 (10 μM) for 90 min at 45 °C and analyzed as in (B). Positions and size of molecular weight markers are shown in kDa and bands corresponding to the respective recombinant proteins are indicated with arrows and labels. Total amounts of proteins in lanes containing the soluble (SF) and insoluble fractions (ISF) were quantified using ImageJ software. For each experiment (C–G), raw values were normalized to the total amount of protein (soluble and insoluble), set to 100% and the bar graphs visualize the proportions of SF vs. ISF, with values and error bars representing mean ± S.D. from three independent experiments.

For assessment of general chaperone activity of TXNL1 towards crude lysate proteins we prepared such from three commonly used cell lines (HEK293T, A549 and AGS). First we evaluated the endogenous expression levels of TXNL1, Trx1 and TrxR1 as well as that of another known small and ATP-independent chaperone, HSP27, which is not a Trx-fold protein but can act as a chaperone in its oligomeric state [39]. This first expression analysis revealed that A549 cells had higher levels of all four analyzed proteins as compared to AGS and 293T, but a basal expression of TXNL1 could be found in all the cell lines while HSP27 could not be detected in HEK293T cells (Fig. 10A). Subsequently we assessed the capacity of HSP27, Trx1 and TXNL1 to act as general chaperones for bulk proteins in cell lysates. Aggregation of cell lysate proteins can easily be induced by incubation at 45 °C, with the insoluble protein fractions thereafter pelleted by centrifugation and analyzed using SDS-PAGE. Thus, a temperature dependent aggregation can be monitored as evident by a comparison to the much lower amount of precipitation formed after incubation at 4 °C (Fig. 10B). As expected, upon addition of pure HSP27 to the HEK293T lysate before heating to 45° prevented the aggregation of cellular proteins in a concentration dependent manner (Fig. 10C). This phenomenon, typical of a chaperone, could not be seen with Trx1 (Fig. 10D). However, it was clearly seen with TXNL1 (Fig. 10E). Similar results were obtained for the A549 and AGS cell lysates, with TXNL1 clearly acting as a chaperone but with less potency as such compared to HSP27 in this assay, while Trx1 completely lacked this activity (Fig. 10F and G).

## 4 Discussion

Here we discovered that TXNL1 has two inherent activities – it is a substrate for TrxR1 and can reduce disulfide substrates through its conserved active site motif shared with Trx1, while in contrast to Trx1 we here found that TXNL1 is also a chaperone. The chaperone activity of TXNL1 does not require its redox activity nor any disulfide-dithiol exchange reactions, as demonstrated using Cys-to-Ser mutant variants. It also did not require ATP for its chaperone activity. With TXNL1 previously shown to interact with the proteasome, these findings suggest that TXNL1 can provide a direct link between the TrxR1-dependent Trx system and diverse mechanisms for regulated protein homeostasis.

As an oxidoreductase, TXNL1 has previously been found to be able to reduce insulin [14] and also disulfides in the PRL protein [40]. In the latter case it is interesting that Trx1 was a worse reductase for PRL than TXNL1, and for efficient PRL reduction, the PITH domain of TXNL1 had to be maintained and was thought to bind directly to PRL [40]. As we here determined the kinetic parameters for TrxR1-mediated reduction of insulin, cystine and GSSG coupled to either TXNL1 or Trx1, it became clear that TXNL1 had much less efficacy in these assays compared with Trx1. With the main reason for the lower efficacy of TXNL1 being at least one order of magnitude higher K_m_ of TrxR1 for TXNL1 than for Trx1, it can be assumed that the additional PITH domain, or the differences in charge distribution and hydrophobicity of TXNL1 compared to Trx1, makes TXNL1 a less efficient substrate for TrxR1. However, with the *k*_*cat*_ values being similar between TXNL1 and Trx1 in these assays, it is clear that TXNL1 has the inherent capacity of acting as an oxidoreductase. With that activity also being fully dependent on the typical CGPC active site motif shared with Trx1, TXNL1 should indeed be recognized as another Trx-fold family protein that can support redox reactions propelled by TrxR1.

The chaperone activity of TXNL1 was not dependent upon its redox activity. This was demonstrated when the redox inactive TXNL C34,37S double mutant or the completely Cys-less TXNL CS variant was added to the Trx-mediated insulin reduction assay. The TXNL1 variants prevented insulin precipitation although none of these variants could reduce insulin directly. We also found that wildtype TXNL1 as well as the Cys-mutated variants directly bound to insulin in the gel filtration experiments, thus keeping insulin in solution by forming larger complexes. Trx1 clearly did not have any of these properties. These observations suggest that the chaperone activity of TXNL1 is mainly independent from its redox activity, or from any of its cysteine residues. It should also be noted that no ATP was included in any of these assays. Thus, we assume that TXNL1 mainly exerts its chaperone activity through non-covalent, protein-protein interactions, which should likely be mediated by the PITH domain. The TEM images further demonstrated the strong potency of TXNL1 to act as a chaperone, in contrast to Trx1, as did its prevention of heat-induced precipitation of crude protein lysates. Although the Cys residues of TXNL1 were not required for the chaperone activities of the enzyme, it can not be ruled out that disulfide-dithiol exchange, or the redox activity of TXNL1, might still regulate its chaperone functions at least in a cellular context. This possibility should preferably be evaluated in forthcoming studies.

Most chaperone foldases, and many additional heat shock proteins, interact with their substrate proteins through a hydrophobic binding domain and require ATP [41,42]. Some redox regulated ATP-independent holdase chaperones have also been well characterized, such as eukaryotic 2-Cys peroxiredoxins or bacterial Hsp33, which become converted to chaperones upon the oxidation of key Cys residues in these proteins [2,43]. In that context it is interesting that we here found that TXNL1, a redox active Trx-fold protein, did not require its Cys residues for its chaperone activity at least as studied herein. It is possible, however, that in a cellular context TXNL1 may provide a redox-mediated link between the Trx system and protein homeostasis. It should also be important to study how the dual activities of this protein may be related to its presumed functions at the proteasome.

## Funding

The project was implemented with the support from the National Research, Development and Innovation Fund of the 10.13039/100020821Ministry of Culture and Innovation under the National Laboratories Program (National Tumor Biology Laboratory (2022-2.1.1-NL-2022-00010)), the Hungarian Thematic Excellence Program (under project TKP2021-EGA-44), and by grant ED_18-1-2019-0025 provided by the National Research, Development and Innovation Office. The project was also funded by 10.13039/501100004047Karolinska Institutet, The Knut and Alice Wallenberg Foundations (KAW 2019.0059), the 10.13039/501100002794Swedish Cancer Society (21 1463 Pj), and the 10.13039/501100004359Swedish Research Council (2021–02214).

## Declaration of competing interest

The authors declare that they have no known competing financial interests or personal relationships that could have appeared to influence the work reported in this paper.

## Data Availability

Data will be made available on request.

## Footnotes

1 See http://www.treefam.org/family/TF314399#tabview=tab1 and https://www.genecards.org/cgi-bin/carddisp.pl?gene=TXNL1&keywords=txnl1 for further details on expression and evolutionary conservation of TXNL1.

2 See https://www.ebi.ac.uk/interpro/entry/InterPro/IPR010400/.
